# Nanoparticle-Enhanced Surface Plasmon Resonance Imaging Enables the Ultrasensitive Detection of Non-Amplified Cell-Free Fetal DNA for Non-Invasive Prenatal Testing

**DOI:** 10.1021/acs.analchem.1c04196

**Published:** 2021-12-29

**Authors:** Marzia Calcagno, Roberta D’Agata, Giulia Breveglieri, Monica Borgatti, Noemi Bellassai, Roberto Gambari, Giuseppe Spoto

**Affiliations:** † Department of Chemical Sciences, University of Catania, Viale Andrea Doria, 6, 95125 Catania, Italy; ‡ Department of Life Sciences and Biotechnology, University of Ferrara, Via Fossato di Mortara 74, 44121 Ferrara, Italy; § INBB, Istituto Nazionale di Biostrutture e Biosistemi, Viale Delle Medaglie D’Oro, 305, 00136 Roma, Italy

## Abstract

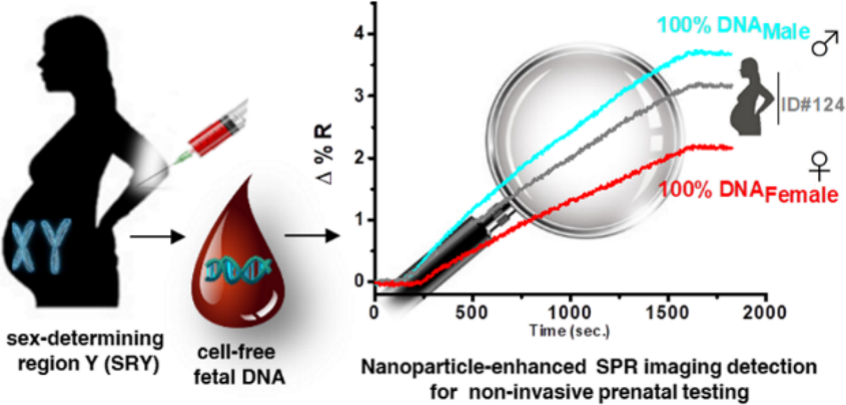

Although many potential applications in early clinical diagnosis have been proposed, the use of a surface plasmon resonance imaging (SPRI) technique for non-invasive prenatal diagnostic approaches based on maternal blood analysis is confined. Here, we report a nanoparticle-enhanced SPRI strategy for a non-invasive prenatal fetal sex determination based on the detection of a Y-chromosome specific sequence (single-gene SRY) in cell-free fetal DNA from maternal plasma. The SPR assay proposed here allows for detection of male DNA in mixtures of 2.5 aM male and female genomic DNAs with no preliminary amplification of the DNA target sequence, thus establishing an analytical protocol that does not require costly, time-consuming, and prone to sample contamination PCR-based procedures. Afterward, the developed protocol was successfully applied to reveal male cell-free fetal DNA in the plasma of pregnant women at different gestational ages, including early gestational ages. This approach would pave the way for the establishment of faster and cost-effective non-invasive prenatal testing.

Prenatal diagnosis and screening of genetic diseases derived from structural and chromosomal anomalies and point mutations are advancing at an unprecedented rate thanks to the recent progress in molecular technologies.^1^ Nowadays, technologies such as chromosomal microarray analysis (CMA),^2^ next-generation sequencing (NGS),^3^ quantitative polymerase chain reaction (qPCR) and fluorescence in situ hybridization (FISH)^4^ are employed to analyze clinical samples collected by invasive methods (i.e., chorionic biopsy or amniocentesis) imposing a small but substantial risk of miscarriage.^5^ Such technologies rely on laborious and time-consuming procedures for the pre-analytical treatment of the biological sample. The discovery of cell-free fetal DNA (cffDNA) in the plasma and serum of pregnant women^6^ paved the way for the non-invasive evaluation of fetal DNA and introduced a breakthrough in non-invasive prenatal testing (NIPT)^7−9^ (Figure 1a). cffDNA represents a small fraction of the total DNA circulating in maternal blood, where cell-free maternal DNA (cfmDNA) is also present. The cffDNA expression level in maternal blood depends on the gestational age and other factors, including maternal weight and fetal aneuploidy.^10^ Such features make NIPT a challenging task demanding to adopt sensitive and specific analytical methods.

**Figure 1:**
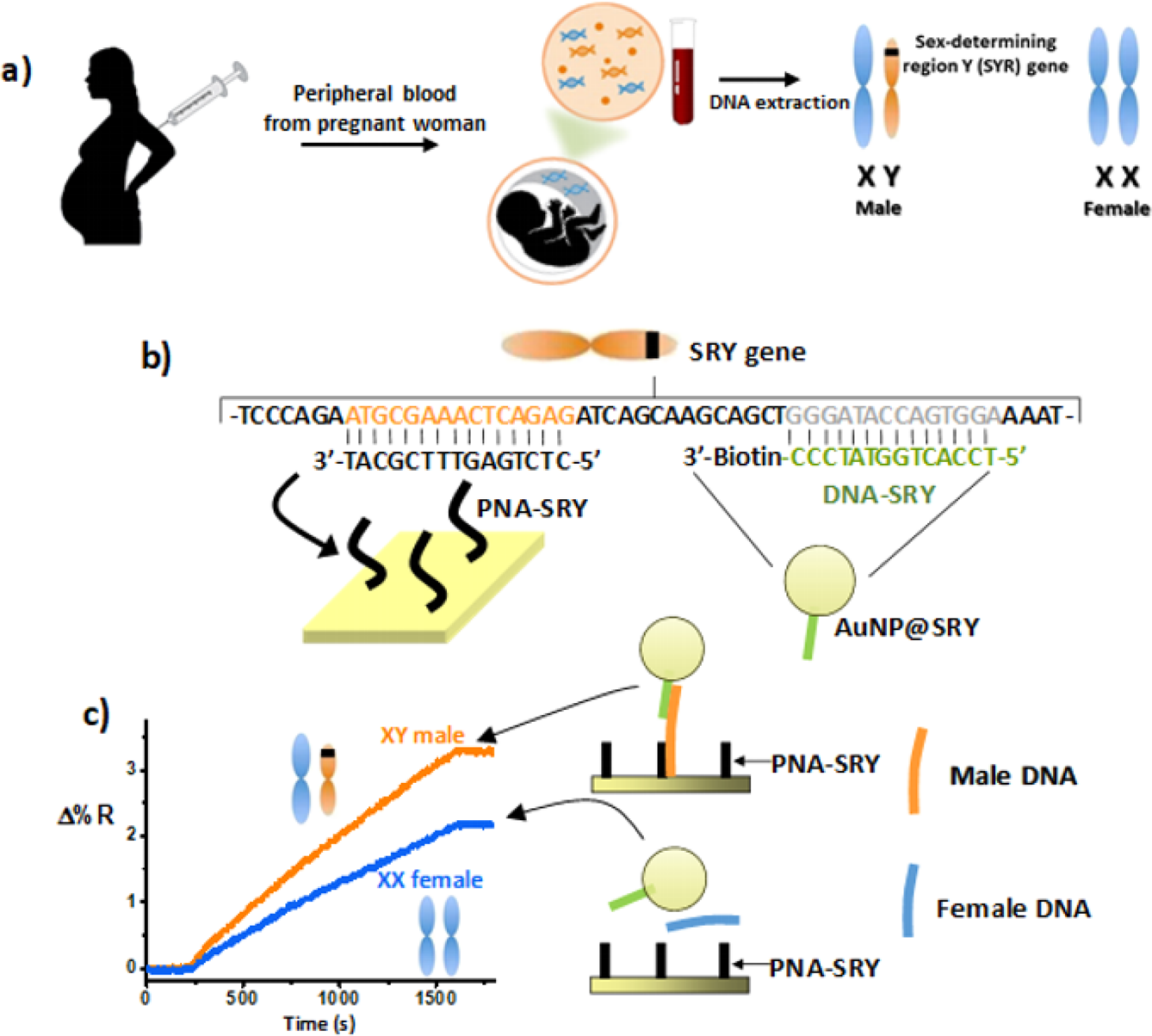
Pictorial description of (a) non-invasive fetal sex determination through a simple blood sampling from a pregnant woman. (b) Human SRY gene located on the Y-chromosome is shown with the sequence targeted by the PNA-SRY probe. The sequence of DNA-SRY biotinylated oligonucleotide (green) immobilized on gold nanoparticles (AuNP@SRY) is also shown. (c) Pictorial description of the sandwich assay for the nanoparticle-enhanced SPRI detection of the SRY sequence.

Currently available technologies allow detection of single-gene disorders by NIPT,^11,12^ although this is still not feasible in a timely and cost-effective way.^10^ NIPT is also adopted for fetal sex determination using PCR-based analytical approaches.^13,14^ Prenatal determination of fetal sex is clinically indicated for pregnancies at risk for genetic disorders affecting a particular sex.^13,15,16^ Those include pregnancies from women carrying X-linked genetic disorders such as adrenoleukodystrophy (ALD) and Duchenne muscular dystrophy (DMD) or hemophilia. In such cases, the fetal sex may guide decisions about invasive testing or specific procedures for managing labor and delivery. The sex-determining region Y (SRY) is not present in the woman genome (Figure 1a). Consequently, the detection of the SRY sequence released by a male fetus in maternal blood is relatively free from interferences caused by cfmDNA and maternal genomic DNA (gDNA). Multiple reports have demonstrated the targeting of Y-chromosome-specific genes for fetal sex determination^17^ and evaluation of risks for sex-linked genetic disorders.^18^ Both single-copy SRY and multi-copy sequences on the Y-chromosome, such as DYS,^14^ can be detected with PCR-based methods for sex determination based on cffDNA. However, false-negative and false-positive results are a matter of concern. Diagnostic accuracy is variable, with sensitivity and specificity ranging from 31 to 100%.^16^ Adopting the SRY gene results in high specificity but low sensitivity for fetal sex since female fetuses are not detected directly but only inferred by a negative result for SRY sequence detection.^19^ False-negative results could also derive from undetectable levels of cffDNA and by PCR amplification of sequences of female DNA with high homology with Y-chromosome.^20^ While conventional PCR-based methods cannot detect cffDNA at levels <20%, a digital droplet PCR (ddPCR) succeeded in cffDNA analysis.^21^ Unfortunately, DNA detection methods relying on the PCR are costly, challenging to parallelize, and prone to sample contamination.^22^

SPR^23^ and SPRI sensors^24^ have been used to investigate an extensive range of biomolecular interactions and applied in many different fields, such as genetically modified organisms (GMOs),^25^ point mutation disorders,^26^ and food safety.^27^ In particular, efforts have been made to develop innovative and ultrasensitive SPR assays for DNA detection not dealing with the PCR amplification of the target sequence to overcome limitations and constraints suffered by PCR-based methods.^28^ In this respect, the signal enhancement by metallic nanoparticles brought a new dimension to SPRI technology by providing high analytical sensitivity through the correct tuning of the plasmonic properties of oligonucleotide-conjugated gold nanoparticles.^29,30^ Nanoparticle-enhanced SPRI assays can take advantage of peptide nucleic acid (PNA) probes to enhance sequence selectivity compared to oligonucleotide probes. The implementation of a sandwich assay configuration using PNA probes has been proven to push the analytical sensitivity of the nanoparticle-enhanced SPRI platform to attomolar concentrations of genome equivalent.^26^

Here, we describe a new nanoparticle-enhanced SPRI assay for the prenatal determination of fetal sex based on the detection of the SRY sequence in cffDNA (Figure 1b,c). The assay does not require the PCR amplification of the target sequence, thus simplifying cumbersome, time-consuming, and error-prone pre-analytical procedures and minimizing the risk of sample contamination compared to state-of-the-art PCR-based assays. The SPRI assay, which shares with conventional qPCR some steps of the pre-analytical treatment of the sample, analyzes the pre-treated sample (Figure 1c) in about 70 min compared with about 210 min needed for qPCR. The nanoparticle-enhanced SPRI method can detect male DNA in mixtures of 2.5 aM male (gDNA_M_) and female (gDNA_F_) genomic DNAs (gDNA). We show that our approach correctly detects male cffDNA in the plasma of pregnant women, thus demonstrating a new application of plasmonic detection with potential in the field of NIPT. To further compare performances of the SPRI approach with the state-of-the-art qPCR, we explored the nanoparticle-enhanced SPRI capacity to detect cffDNA isolated from maternal plasma obtained from pregnant women at early gestational age (5–10 weeks). We show that the SPRI assay overcomes the already described difficulty of qPCR in identifying male fetal sex at early gestational stages.^21^

## Experimental Section

### Reagents and Materials

Reagents were obtained from commercial suppliers and used without further purification. Wild-type streptavidin (WT-SA) was purchased from Invitrogen (Italy). Nitrocellulose membrane filters were purchased from Whatman (U.K.). Trisodium citrate dihydrate, tetrachloroauric(III) acid, ethanol, dimethyl sulfoxide, sodium hydroxide solutions (10 M in water), and dithiobis(*N*)succinimidylpropionate (DTSP) were purchased from Merck (Italy). Phosphate-buffered saline (PBS) solutions at pH 7.4 (137 mM NaCl, 2.7 mM KCl, and 10 mM phosphate-buffered saline were obtained from Amresco (Italy). Oligonucleotides were purchased from Thermo Fisher Scientific, Inc. (Italy). The PNA-SRY probe (Table 1) was purchased from Panagene Inc. (South Korea). SPRI gold chips were purchased from Xantec Bioanalytics (Germany). Ultrapure water (Milli-Q Element, Millipore) was used for all the experiments.

**Table 1:** Oligonucleotide and PNA Sequences and Acronyms

acronym	sequence	*T*_m_ (°C)
DNA-SRY	3′-biotin-CCCTATGGTCACCT-5′	44.0
PNA-SRY	(AEEA)_2_CTCTGAGTTTCGCAT^a^	67.2

a AEEA: {2-[2-(2-amino-ethoxy)-ethoxy]-ethoxy}-acetic acid.

### Blood Sample Collection and Plasma Isolation

Blood samples from 19 pregnant women (gestational age ranging from 5 to 37 weeks, 15 male-bearing pregnancies) or from healthy donors were drawn in test tubes containing EDTA after approval by the Ethical Committee of University Hospital S. Anna, Ferrara (Italy). Informed consent was obtained from all subjects, and we conducted experiments in agreement with the Declaration of Helsinki. Each specimen was identified with a progressive number (# ID) to ensure donors’ anonymity. Depending on whether samples were used to prepare plasma for circulating DNA extraction (pregnant women) or to extract genomic DNA (healthy donors), they were promptly processed or stored at −80 °C until use, respectively. According to the protocol elsewhere described, we isolated plasma within 3 h from blood collection.^31^ We homogenized blood samples using a tube roller mixer (5–10 min); then, we centrifuged them at 1200*g* for 10 min at 4 °C without brake. Plasma was then carefully collected and centrifuged again (2400*g* for 20 min at 4 °C) to remove platelets and precipitates. The resulting supernatant was collected and stored at −80 °C into single-use aliquots.

### Extraction of Circulating Cell-Free DNA (cfDNA) and PCR Quantification

cfDNA was extracted from 2 mL of maternal plasma, not thawed more than once, using the QIAamp DSP Virus Spin kit (Qiagen, Hilden, Germany), according to the manufacturer’s instructions. DNA elution was performed in 60 μL of Amp Viral RNA Elution (AVE) buffer. Six microliters of circulating DNA extracted from maternal plasma was analyzed by real-time qPCR assays specific for the β-globin gene (forward: 5′-GCAAAGGTGCCCTTGAGGT-3′; reverse: 5′-CAAGAAAGTGCTCGGTGCCT-3′; β-globin probe: 5′-FAM/TAGTGATGG/ZEN/CCTGGCTCACCTGGAC/3IABkFQ-3′), and for the SRY gene (forward: 5′-CCCCCTAGTACCCTGACAATGTATT-3′; reverse: 5′-TGGCGATTAAGTCAAATTCGC-3′; SRY probe: 5′-FAM/AGCAGTAGA/ZEN/GCAGTCAGGGAGGCAGA/3IABkFQ-3′), to quantify the total and (in case of a male fetus) fetal cfDNA, respectively. Every reaction containing TaqMan Universal PCR Master mix (Thermo Fisher Scientific Inc.) had a final volume of 15 μL and was performed in duplicate. For each analysis, standard male genomic DNAs were prepared to get known amounts of DNA corresponding to 10, 25, 50, and 100 copies of the SRY gene, and 200, 1000, 2000, and 10,000 copies of the β-globin gene, respectively, and to make a calibration line for the absolute quantification of samples. No template controls were included as well. The reactions were carried out on a StepOne real-time PCR system (Applied Biosystems, Life Technologies) using the StepOne Software, v2.3 (Applied Biosystems, Thermo Fisher Scientific Inc.) and the following amplification program: 2 min at 50 °C, 10 min at 95 °C, 50 amplification cycles comprising a denaturation step at 95 °C for 15 s, and an annealing–elongation step at 60 °C for 1 min.

### Functionalized Gold Nanoparticles (AuNP@SRY)

We synthesized^30^ gold nanoparticles (AuNPs) with citrate reduction of HAuCl_4_·3H_2_O and characterized them with UV–vis spectroscopy (Agilent 8453 spectrometer) and transmission electron microscopy (TEM, Jeol JEM-2000 FX II, operating at 200 kV). We conjugated them to the DNA-SRY oligonucleotide (Table 1) according to a procedure elsewhere described^32^ to obtain AuNP@SRY. The concentration of AuNP@SRY stock solutions (typically 1–3 nM) was obtained with UV–vis spectroscopy (ε_528_ = 2 × 10^8^ M^–1^ cm^–1^).^32^ We used 0.1 nM (in PBS) AuNP@SRY dispersions for the nanoparticle-enhanced SPRI experiments.

### Nanoparticle-Enhanced SPRI

We performed SPRI experiments at room temperature using an SPR imaging apparatus (GWC Technologies, USA) and analyzed the acquired images with V++ (version 4.0, Digital Optics Limited, New Zealand) and ImageJ 1.32j (National Institutes of Health, USA) software packages. SPRI data (pixel intensity, 0–255 scale) was converted into the percentage of reflectivity (%R) using the equation %R = 100 × 0.85*I*_p_/*I*_s_ (*I*_p_ and *I*_s_ refer to the reflected light intensity detected using p- and s-polarized light, respectively). We obtained kinetic data by plotting the difference in %R (Δ%R) from selected regions of interest of SPR images as a function of time. A poly(dimethylsiloxane)-based microfluidic device with six parallel microchannels (80 μm depth, 1.4 cm length, and 400 μm width) provided independent control of interactions occurring at six different regions of the gold chip. We fabricated it by replica molding^33^ and used PEEK tubes (UpChurch Scientific) inserted into the device to connect it to an Ismatec IPC (Ismatec SA, Switzerland) peristaltic pump. The statistical treatment of data was performed using the computer software OriginPro, version 9.0 (OriginLab Corporation, Northampton, USA).

### PNA Probe Sequence Design and Surface Immobilization

The PNA probe sequence (PNA-SRY, Table 1) was designed to have a high melting temperature (higher than 65 °C at 4 μM strand concentration), as calculated according to an empirical model.^34^ BLAST^35^ searches were performed to ensure that the PNA-SRY targeted sequence in the SRY gene is not homologous to other sequences in gDNA. We immobilized PNA-SRY on DTSP-functionalized gold chips obtained after the immersion of clean gold chips in a DTSP solution (4 mM in DMSO, 48 h, 23 °C) kept under constant and gentle agitation. The modified chip was then thoroughly rinsed with ethanol. The PNA-SRY probe was immobilized through the amine-coupling reaction between *N*-hydroxysuccinimidyl ester ends of DTSP and the N-terminal group of an (AEEA)_2_ linker (Table 1) present at the N-terminus of PNA-SRY. The (AEEA)_2_ linker increases the accessibility of target DNA.^36^ The spatially separated immobilization of the PNA-SRY probe was obtained by injecting PNA-SRY solutions (0.1 μM in PBS, a flow rate of 10 μL min^–1^) into the parallel channels of the microfluidic device in contact with the DTSP-modified gold surface at a density of 3 × 10^12^ molecules cm^–2^ (details on the probe density calculations are reported in the Supporting Information).

### DNA Pre-Analytical Treatment and SPRI Experiments

Before SPRI analyses, gDNAs or cfDNA isolated from plasma samples of pregnant women were fragmented by sonication (3 min, ELMA Transsonic T480/H-2) and vortexing (1 min, IKA Vortex GENIUS 3) and denatured by heating at 95 °C for 5 min. We used gDNA_F_ (female DNA) and gDNA_M_ (male DNA) as negative and positive controls, respectively. The strands’ reassociation before SPRI detection was prevented by cooling samples on ice (1 min) before introducing them into the SPRI microfluidics apparatus. gDNA_M_ (sample from a donor, sample ID = 58P; stock solution = 75.50 ng μL^−1^) and gDNA_F_ (sample from a donor, sample ID = LC; stock solution = 31.27 ng μL^−1^) were mixed and used as proxies for cffDNA in pregnant women bearing a male fetus.

## Results and Discussion

### Nanoparticle-Enhanced SPRI Identifies Non-Amplified Male and Female gDNA

**Figure 2:**
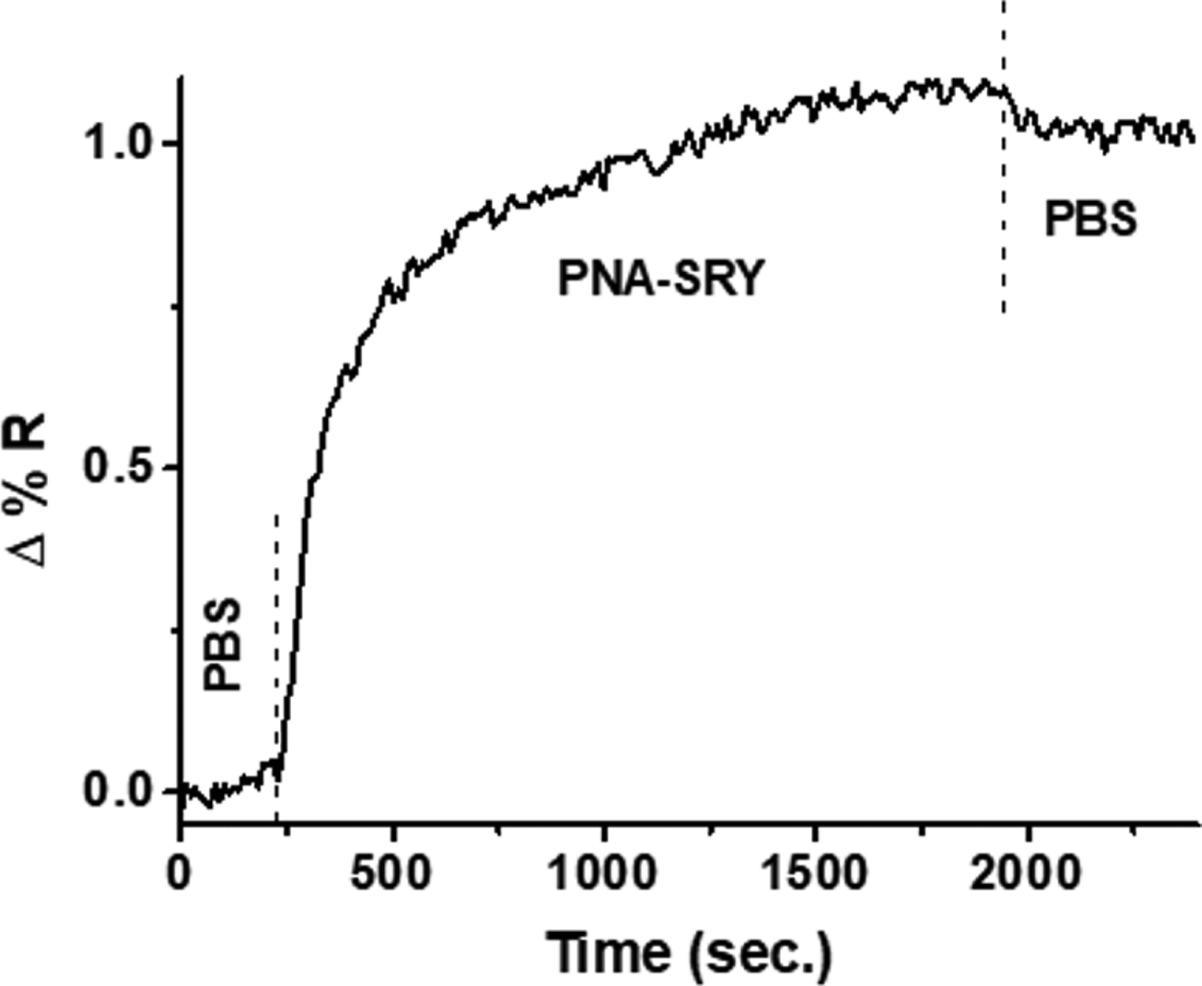
Representative changes in percent reflectivity (Δ%R) over time detected for the immobilization of the PNA-SRY probe.

To select optimal experimental conditions and test our nanoparticle-enhanced SPRI assay’s performances, we performed proof-of-concept measurements analyzing male and female gDNAs. In particular, we analyzed 5 pg μL^–1^ solutions (in PBS) of gDNA_F_ and gDNA_M_ isolated from the female and male donors’ blood. We selected this specific concentration value to simulate conditions for cffDNA in maternal plasma at the early gestational stage. We treated samples as described and performed the nanoparticle-enhanced SPRI detection with no PCR amplification of the genetic sample. The nanoparticle-enhanced SPRI assay is based on a sandwich detection approach involving three steps. The PNA-SRY probe is first immobilized on the sensor surface (Figure 2). The gDNA sample is then adsorbed on the functionalized surface. PNA-SRY hybridizes with the complementary sequence in the SRY gene of gDNA_M_, whereas no specific interaction is established with gDNA_F_.

**Figure 3:**
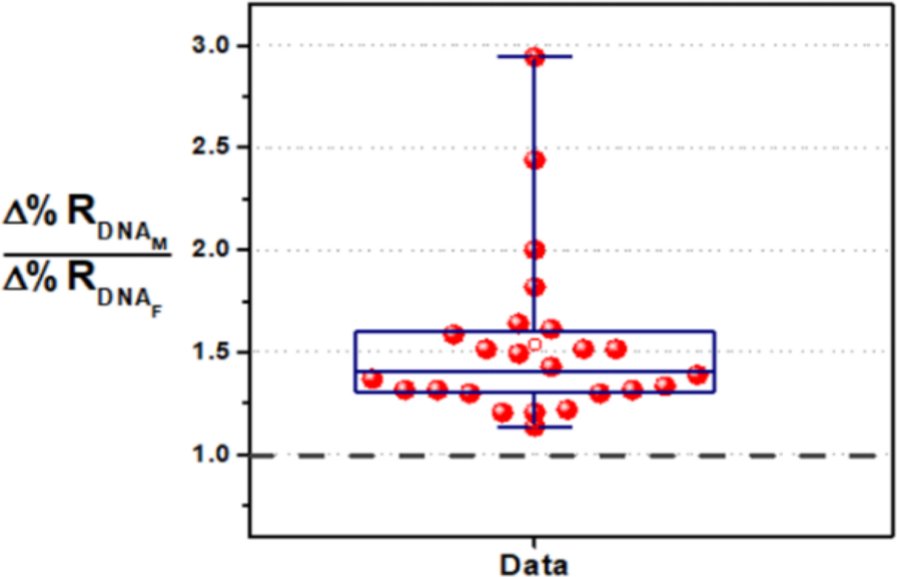
Δ%RDNA_M_/Δ%RDNA_F_ values for the analysis of gDNAs isolated from the blood of male and female donors. We analyzed female and male gDNA samples in parallel and considered the ratio of %R values detected after the adsorption of the same AuNP@SRY dispersion on surfaces resulting from the interaction of gDNA_M_ and gDNA_F_ samples, respectively. Male samples produced higher SPRI signals than female samples due to the preferential interaction of gDNA_M_ with PNA-SRY. PNA-SRY is complementary to the target sequence in the SRY gene. A black dashed line is shown to better identify Δ%RDNA_M_/Δ%RDNA_F_ values greater than 1.

AuNP@SRY are introduced in the assay’s last step to enhance plasmonic detection (Figure 1c). AuNP@SRY are functionalized with an oligonucleotide (DNA-SRY) whose sequence is complementary to a portion of the gDNA_M_ sequence not hybridized with PNA-SRY. Figure 2 shows representative changes in percent reflectivity (Δ%R) over time detected for PNA-SRY immobilization through amine coupling with the DTSP-modified gold surface (0.1 μM in PBS, flow rate of 10 μL min^–1^; immobilized probe density is 3 × 10^12^ molecules cm^–2^). We used microfluidic devices bearing parallel microchannels to independently functionalize different sensor surface areas to analyze up to six different samples simultaneously. The SPRI signal we detected for the adsorption of gDNA_M_ or gDNA_F_ (5 pg μL^–1^, flow rate of 10 μL min^–1^) was close to the instrumental noise and not helpful to evaluate the correct interaction between the genetic sample and PNA-SRY probe. The surface was then washed with PBS for at least 30 min to desorb most non-specifically adsorbed molecules from the sensor surface. After AuNP@SRY enhancement (0.1 nM in PBS, flow rate of 10 μL min^–1^), the assay discriminated between gDNA_M_ and gDNA_F_ samples and highlighted the preferential interaction of gDNA_M_ with PNA-SRY (Figure 1c). The signal detected for gDNA_F_ after AuNP@SRY enhancement is influenced by non-specifically adsorbed DNA fragments that can trigger nanoparticle accumulation on the surface due to the alteration of the local charge balance they cause, as elsewhere discussed.^30^ A similar phenomenon has also been observed when the experimental conditions required for the nanoparticle-enhanced SPRI ultrasensitive detection of nucleic acid sequences were implemented with an antifouling surface layer.^37^ Considering the non-specific AuNP@SYR adsorption’s possible contribution to the detected SPRI signals, we calculated the ratio between signals detected from the two surfaces (i.e., gDNA_M_ and gDNA_F_). Specific interaction with PNA-SRY is possible only for gDNA_M_, whereas similar non-specific contributions are provided by both gDNA_M_ and gDNA_F_. Fluctuations in the SPRI absolute signal produced by different AuNP@SRY dispersion batches are typically observed due to the exceptional sensitivity of SPRI to AuNPs. For this reason, we performed experiments analyzing the male and female genetic samples in parallel and considered the ratio of SPRI responses (Δ%R) detected after the adsorption of the same AuNP@SRY dispersion on the surfaces resulting from PNA-SRY interaction with gDNA_M_ and gDNA_F_ samples, respectively (Figure 3).

Figure 3 shows Δ%RDNA_M_/Δ%RDNA_F_ values referred to the replicated analyses of gDNAs isolated from the blood of male (gDNA_M_) and female (gDNA_F_) donors. As expected, male samples produced higher SPRI signals than female samples, resulting in values of Δ%RDNA_M_/Δ%RDNA_F_ ratio greater than 1 (Figure 3), thus demonstrating the assay capacity to detect male or female DNAs correctly.

### Nanoparticle-Enhanced SPRI Detects Non-Amplified Male gDNA in Male/Female gDNAs Mixtures

**Figure 4:**
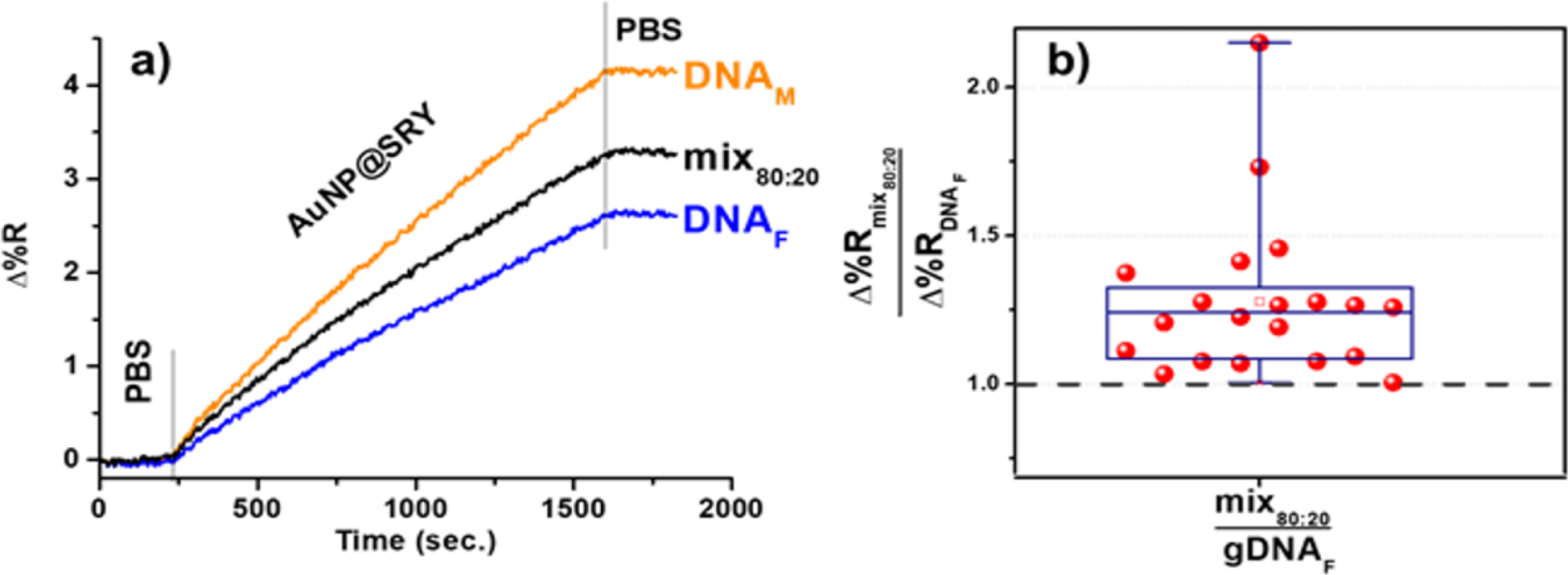
(a) Representative changes in percent reflectivity (Δ%R) over time detected for the parallel adsorption of AuNP@SRY (0.1 nM in PBS) on gDNA_F_ (female donor, blue line), mix_80:20_ (proxy of cffDNA in a pregnant woman bearing a male fetus, black line), and gDNA_M_ (male donor, orange line), respectively. Before AuNP@SRY adsorption, each sample was adsorbed on a surface-immobilized PNA-SRY probe (5 pg μL^–1^, flow rate of 10 μL min^–1^). (b) Δ%Rmix_80:20_/Δ%RDNA_F_ values for 20 replicated experiments for the parallel analysis of gDNAs isolated from the blood of female donor (gDNA_F_) and mix80:20. A black dashed line is shown to identify Δ%Rmix_80:20_/Δ%RDNA_F_ values larger than 1.

Having demonstrated our nanoparticle-enhanced SPRI assay’s analytical capacity on male and female gDNAs, we moved to mixtures of male and female gDNAs. We used such mixtures as proxies for cffDNA in pregnant women bearing a male fetus. cffDNA is a fraction of the total cfDNA in maternal blood, and its percentage is dynamically modulated and influenced by several factors, including gestational age, maternal weight, and fetal aneuploidy. cffDNA percentage values follow a normal-like distribution centered between 10 and 20% at gestational age comprised between 10 and 21 weeks.^10^ On this basis, we prepared 80:20 mixtures of gDNA_F_ and gDNA_M_ (mix_80:20_) to evaluate the capacity of our nanoparticle-enhanced SPRI method to discriminate between the mix (proxy for a pregnant woman bearing a male fetus), gDNA_F_ (female donor), and gDNA_M_ (male donor) samples. We used the nanoparticle-enhanced SPRI assay as described and detected the mix_80:20_, gDNA_M_, and gDNA_F_ samples in parallel. Figure 4a shows representative SPRI responses detected for the parallel adsorption of AuNP@SRY on three surfaces resulting from the interaction of the genetic samples (gDNA_M_, mix_80:20_, and gDNA_F_) with PNA-SRY. The total DNA concentration in each sample was 5 pg μL^–1^ (∼2.5 aM, assuming 3.2 × 10^9^ nucleotides in gDNA and MW = 2.1 × 10^12^). Nanoparticle-enhanced SPRI discriminated the samples with mix_80:20_ providing a signal typically between those detected for gDNA_M_ and gDNA_F_, respectively. In any case, mix_80:20_ provided higher SPRI signals than gDNA_F_. Figure 4b summarizes results we obtained from 20 independent experiments performed by analyzing in parallel the different samples (i.e., gDNA_F_ and mix_80:20_). We referred SPRI signal detected for mix_80:20_ to the signal detected for gDNA_F_ in parallel and calculated the ratio of signals by considering Δ%R after 1500 s of AuNP@SRY adsorption. As expected, Δ%Rmix_80:20_/Δ%RDNA_F_ values were greater than 1 (i.e., mix_80:20_ samples produced more intense SPRI signals compared to gDNA_F_, 95% confidence interval for the mean 1.28 ± 0.12, *n* = 20), thus demonstrating the assay capacity to correctly detect the proxy for a pregnant woman bearing a male fetus.

SPRI kinetics curves for the interaction of AuNP@SRY with surfaces referring to gDNA_F_, gDNA_M_, and mix_80:20_ samples are shown in the Supporting Information (Figure S1a–x). Table S1 displays Δ%R values from replicated experiments performed by analyzing gDNA_F_, gDNA_M_, and mix_80:20_ samples in parallel. The same data and their ratio values are shown in the box plots reported in Figures S2 and S3. We applied the non-parametric Kruskal–Wallis test to verify that medians of the populations to which Δ%RDNA_M_/Δ%RDNA_F_ and Δ%Rmix_80:20_/Δ%RDNA_F_ samples belong are significantly different (α = 0.05, *p*-value = 0.0016). Values of Δ%RDNA_M_/Δ%RDNA_F_ and Δ%Rmix_80:20_/Δ%RDNA_F_ from replicated experiments were characterized by CV% = 27 and CV% = 20, respectively. The data distribution is mainly influenced by the strong dependence of the measured SPRI signals from characteristics of AuNP@SRY dispersions. The specific assay configuration, using the same AuNP@SRY dispersion for the parallel analysis of the selected sample and gDNA_F_ acting as the negative control, allowed the correct discrimination between samples bearing the SRY target sequence (i.e., male DNA) and female samples (Δ%RDNA_M_/Δ%RDNA_F_ and Δ%Rmix_80:20_/Δ%RDNA_F_ values greater than 1). The assay configuration thus made the analysis (i.e., identification of male DNA) not significantly affected by fluctuations caused by variability in AuNP@SRY dispersions.

### Nanoparticle-Enhanced SPRI Identifies Non-Amplified Male cffDNA from Maternal Blood

The results from experiments with gDNAs prompted us to apply the SPRI assay to determine fetal gender detecting cffDNA bearing the SRY sequence and circulating in maternal blood. We extracted cfDNA from the plasma obtained from the blood of eight male and four female-bearing pregnancies with this aim. We preliminarily validated the samples by real-time qPCR (Table 2). Weeks of gestation (wog) for those male- and female-bearing pregnancies ranged between 16 and 37. We initially analyzed samples from wog 16 due to an insufficient cffDNA amount in maternal plasma at earlier gestational age for qPCR analysis.^38,8^ To test the SPRI capacity to detect male cffDNA from early pregnancies, we isolated cffDNA from seven pregnant women’s plasma with wog ranging between 5 and 10. We validated the samples by ddPCR due to the limitation of qPCR in analyzing early pregnancy samples (Table 2). (Details on the ddPCR are reported in the Supporting Information.)

**Table 2:** Pregnant Women Blood Samples with their Assigned **#**ID, Weeks of Gestation and Proportion of Cell-Free Fetal DNA (cffDNA) Carrying the SRY Sequence over the Total cfDNA (Quantified by Real-Time qPCR)^a^

sample ID#	weeks of gestation (wog)	total cfDNA (pg μL^–1^)	cffDNA (pg μL^–1^)	cffDNA/total cfDNA (%)
Male-bearing pregnancy				
107	36	80	8.0	10
112	31	658	18.9	2.9
117	30	1051	13.6	1.3
121	30	2870	91.6	3.2
157	25	393	3.8	0.97
100	21	1240		
124	18	670	9.4	1.4
96	16	98	2.9	2.9
Female-bearing pregnancy				
55	37	144		
37	37	118		
41	29	30		
151	28	471		

Male-bearing early pregnancy^a^				
sample ID#	weeks of gestation (wog)	total cfDNA (copies μL^–1^)	cffDNA (copies μL^–1^)	cffDNA/total cfDNA (%)
277	10	20.6	0.25	1.21
212	8	29.6	0.08	0.27
230	8	19.3	0.66	3.42
190	7	42.3	0.06	0.14
247	7	24.9	0.11	0.44
229	6	9.4	0.20	2.13
218	5	8.2	0.06	0.73

a For samples at early wog, not validated in qPCR, the number of copies μL^–1^ is shown as analyzed by ddPCR.

**Figure 5:**
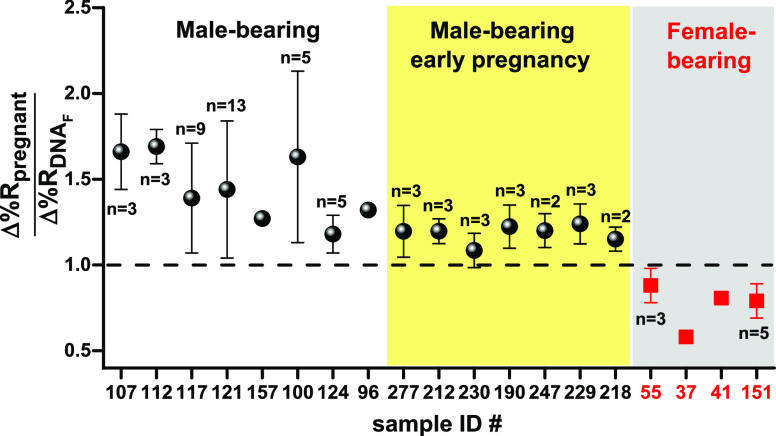
Mean Δ%R_pregnant_/Δ%RDNA_F_ values obtained for the SPRI parallel detection of the target SRY sequence in gDNA_F_ (5 pg μL^–1^) and cffDNA from male-bearing pregnancy (107, 112, 117, 121, 157, 100, 124, and 96) and male-bearing early pregnancy (277, 212, 230, 190, 247, 229, and 218) samples. Δ%R_pregnant_/Δ%RDNA_F_ values are also reported for the parallel detection of gDNA_F_ (5 pg μL^–1^) and cffDNA from female-bearing pregnancy samples (samples 55, 37, 41, and 151; no SRY sequence). The number of replicates (*n*) is shown. The analysis of samples 157, 96, 37, and 41 was not replicated due to limitations in the sample size. Error bars represent the population mean confidence interval at the 95% level. As expected, Δ%R_pregnant_/Δ%RDNA_F_ values for male-bearing pregnancy are greater than 1, thus demonstrating the assay capacity to detect cffDNAs carrying the SRY sequence (i.e., male DNA) correctly.

We analyzed the pregnant women’s samples with SPRI and used gDNA_F_ to conduct parallel control analyses. We referred to the nanoparticle-enhanced SPRI response detected for gDNA_F_, the signal detected from the pregnant woman’s sample (Δ%R_pregnant_). Figure 5 shows mean values of Δ%R_pregnant_/Δ%RDNA_F_ for the analyzed samples.

All samples from a male-bearing pregnancy provided Δ%Rpregnant/Δ%RDNA_F_ values larger than 1, as expected for the ratio of signals referring to samples bearing cffDNA from a male fetus and samples with only female DNA (gDNA_F_). Samples from female-bearing pregnancies provided Δ%Rpregnant/Δ%RDNA_F_ values lower than 1 instead, thus providing evidence of the preferential interaction of AuNP@SRY with surfaces resulting from the adsorption of gDNA_F_ rather than samples from female-bearing pregnancy on PNA-SRY. Two families of cfDNAs circulate in the pregnant woman’s blood: cell-free maternal DNA (cfmDNA) and cffDNA. Both cfmDNA and cffDNA do not carry the SRY sequence for female-bearing pregnancy. However, the relatively small size of cffDNA and the difference in size compared to cfmDNA^39^ may be responsible for the different surface environments obtained after the adsorption of samples on the SPRI-functionalized surface, causing the preferential non-specific adsorption of AuNP@SRY on gDNA_F_. As already pointed out, when DNA fragments are retained non-specifically by the PNA-SRY-modified surface, the adsorption of conjugated AuNP@SRY, driven by non-cross-linking interactions with the surface, is obtained.^25^ Data shown in Figure 5 demonstrates that also samples from male-bearing pregnancies at early gestational weeks provided Δ%R_pregnant_/Δ%RDNA_F_ values larger than 1, thus correctly discriminating between samples bearing cffDNA from a male fetus and samples with only female DNA (gDNA_F_). Such evidence provides a more defined framework for comparing conventional PCR-based methods (i.e., qPCR) and the SPRI PCR-free assay for the non-invasive prenatal sex determination. SPRI assay simplifies the analytical workflow compared to PCR-based methods with an analysis time of about 70 min compared with about 210 min needed for the qPCR. Despite the sensitivity and specificity of the qPCR method, this technique has limitations for diagnosing fetal sex when a reduced amount of template is available due to the insufficient amount of circulating fetal DNA available in maternal plasma at early gestational weeks.^21^ The described SPRI assay correctly identifies male cffDNA in maternal plasma samples at 5 to 10 wog. Therefore, it could be a practical and alternative approach to qPCR for earlier prenatal diagnosis of fetal sex.

## Conclusions

The relatively low amount of cffDNA circulating in maternal blood imposes highly sensitive and specific detection approaches to perform non-invasive prenatal diagnosis for pregnant women. In this way, traditional invasive testing might be avoided, and the risk of a miscarriage of ∼1% might be reduced or eliminated. In the non-invasive prenatal diagnosis (NIPD) field for sex-related diseases, fetal sex determination is commonly performed by targeting Y-chromosome-specific sequences, within the single-copy SRY gene, by a qPCR amplification step^40^ or its innovative approach such as digital PCR,^9,21^ although with potential contaminations and possible false results primarily associated with required procedures.

To our knowledge, a strategy based on SPRI technology has not yet been investigated for detecting cffDNA present in maternal plasma. Here, we described the use of a nanoparticle-enhanced SPRI assay for the molecular identification of a sex-specific gene in the non-amplified human genomic DNA isolated from blood samples of pregnant women. The findings of this study demonstrate that the non-invasive prediction of fetal sex from the examination of cffDNA in maternal blood can be achieved through an SPRI assay targeting the Y-chromosome single-copy sequence. The assay performances demonstrate that the new method offers the opportunity to determine fetal gender detecting cffDNA circulating in maternal blood at an attomolar level with a PCR-free amplification reaction, thus minimizing the risk of sample contamination, preventing biases and artifacts, and reducing assay time compared to conventional PCR-based approaches. In particular, all plasma samples were correctly determined for the SRY gene presence using our SPRI assay even at the earliest gestational age (wog 5–10). Since the capacity of the nanoparticle-enhanced SPRI method to discriminate single-base mutations has been recently demonstrated,^37,41^ a similar approach is envisaged to be applied for non-invasive prenatal diagnostics.

## Supporting Information Available

PNA probe density calculations, SPRI kinetics curves, statistical analysis, and ddPCR (PDF).
