# Supplementary material for: Nanoparticle-Enhanced Surface Plasmon Resonance Imaging Enables the Ultrasensitive Detection of Non-Amplified Cell-Free Fetal DNA for Non-Invasive Prenatal Testing
Source: Anal Chem. 2021 Dec 29;94(2):1118–25. doi: 10.1021/acs.analchem.1c04196 (PMC8771635; doi:10.1021/acs.analchem.1c04196)
Supplement: Supplementary file 1 — ac1c04196_si_001.pdf [file ac1c04196_si_001.pdf]

Supporting Information

Nanoparticle-enhanced surface plasmon resonance imaging enables the ultrasensitive detection of non-amplified cell-free fetal DNA for non-invasive prenatal testing

Marzia Calcagno,[1] Roberta D'Agata,[1*] Giulia Breveglieri,[2] Monica Borgatti,[2] Noemi Bellassai,[1] Roberto Gambari,[2] Giuseppe Spoto[1, 3]

[1] Department of Chemical Sciences, University of Catania, Viale Andrea Doria, 6, 95125, Catania, Italy

[2] Department of Life Sciences and Biotechnology, University of Ferrara, Via Fossato di Mortara 74, 44121 Ferrara, Italy

[3] INBB, Istituto Nazionale di Biostrutture e Biosistemi, Viale Delle Medaglie D'Oro, 305, 00136, Roma, Italy

* Email: **dagata.r@unict.it**

Table of contents

Experimental Section

Figure S1(a-x). SPRI responses in Δ%R values obtained when conjugated AuNP@SRY were adsorbed in parallel on $gDNA_M$, $gDNA_F$ and $mix_{80:20}$ samples previously adsorbed on PNA-SRY.

Figure S2. Data/box plots showing the Δ% R values for AuNP@SRY enhanced detection of $gDNA_M$, $gDNA_F$ and $mix_{80:20}$.

Figure S3. Box/data plots showing values of $\Delta\%RDNA_M/\Delta\%RDNA_F$ and $\Delta\%Rmix_{80:20}/\Delta\%RDNA_F$ ratios.

Table S1. Δ%R values for the AuNP@SRY enhanced parallel detection of $gDNA_M$, $gDNA_F$ and $mix_{80:20}$ samples.

Table S2. Repeatability statistics of the ratio values $\Delta\%RDNA_M$ /$\Delta\%RDNA_F$ and $\Delta\%Rmix_{80:20}/\Delta\%RDNA_F$.

Experimental Section

PNA probe density calculation. PNA-SRY density was estimated based on the model described by Shumaker-Parry et al.[1] Values for the specific density, molecular weight and refractive index of the PNA probe are 1.2 (g/cm^3), 4422 (g/mol) and 1.40, respectively. The refractive index of PBS buffer is 1.33. We also considered the decay length l_d as 37% of the SPR wavelength.[2] The sensitivity factor for the SPRI system was s = 6009.28 %R/RIU.

Binding kinetics curves for the gDNAs hybridization. The direct adsorption of the $gDNA_M$, the $gDNA_F$, the $mix_{80:20}$ and DNA from pregnant women on the PNA-SRY functionalized surface did not generate any SPRI response valid for samples discrimination. The absorption of the genetic samples on the PNA-SRY immobilized surface generated an SPRI signal close to the instrumental noise. During the experiments, temperature fluctuations (in the 0.1-0.3°C range) contributed to the signal instability, and no signal attributable to any specific or non-specific interaction involving the genomic DNA was evident.

Droplet digital PCR. Circulating DNA was detected and quantified by using a QX200™ Droplet Digital™ PCR system (Bio-Rad Laboratories, Hercules, CA, USA). In order to generate the droplets, 20 µL of ddPCR reaction, containing ddPCR Supermix for Probes (Bio-Rad Laboratories), EIF2C1 Bio-Rad assay (ID: dHsaCp2500349, HEX labeled), SRY assay[3] and DNA template, were inserted into an eight-well cartridge, together with 70 µL of Droplet Generation Oil for Probes (Bio-Rad Laboratories), by using an Automated Droplet Generator (Bio-Rad Laboratories) according to the manufacturer's instructions. Then 40 µL of the generated droplet emulsion were transferred to a new 96-well PCR plate and amplified by using the following conditions: 10 min at 95 °C, followed by 45 cycles of a two-step thermal profile of 30 s at 94 °C and 1 min at 60 °C, and finally 10 min at 98 °C. Then plates were analyzed by a QX200™ Droplet Reader (Bio-Rad Laboratories) and the QuantaSoft software (1.3.2.0; Bio-Rad Laboratories).

Figure S1 a-x). SPRI responses in Δ%R values obtained when conjugated AuNP@SRY were adsorbed in parallel on gDNA$_M$, gDNA$_F$ and mix$_{80:20}$ samples previously adsorbed on PNA-SRY probe.

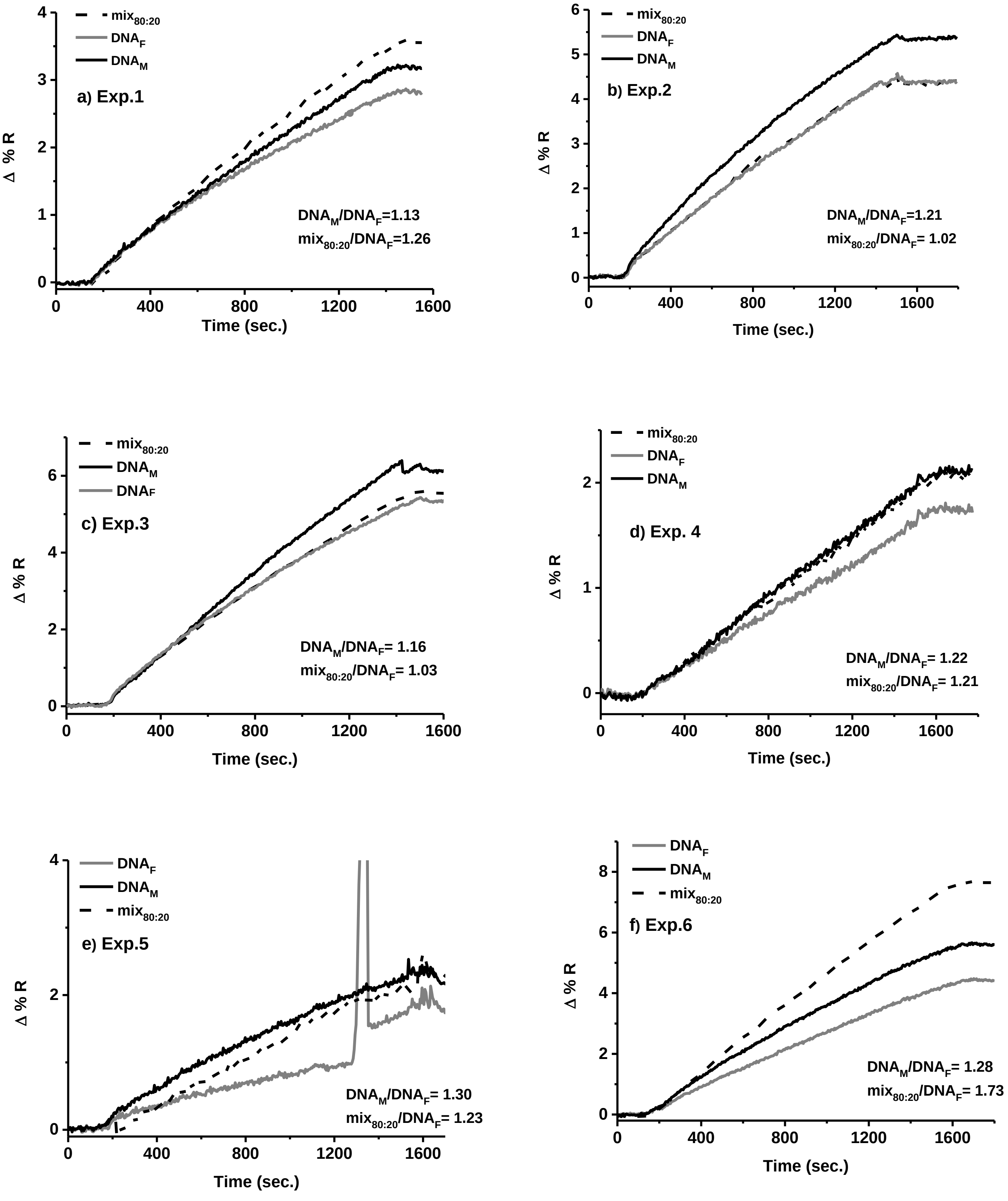

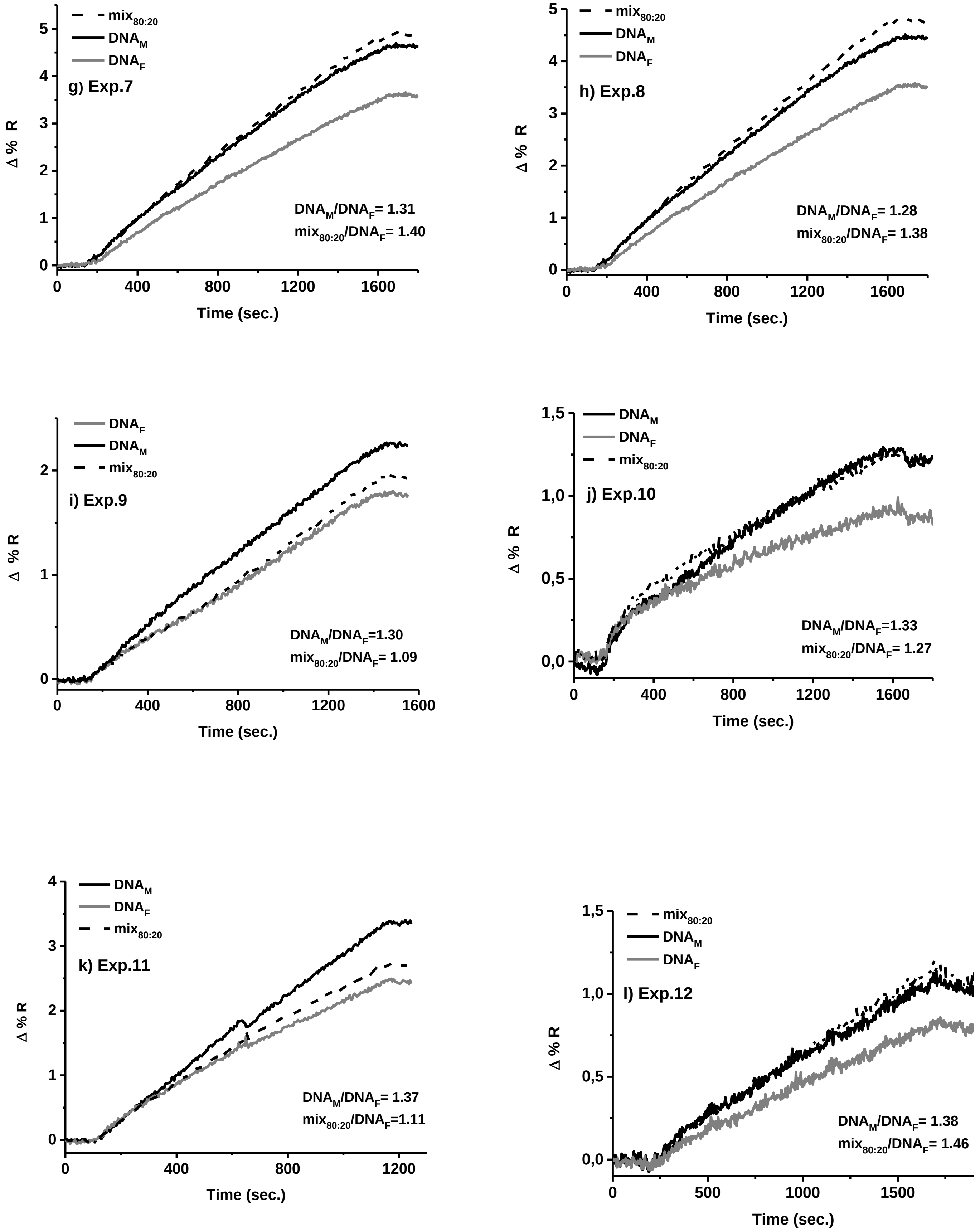
g) Exp.7
$mix_{80:20}$
DNA_M
DNA_F
DNA_M/DNA_F= 1.31
$mix_{80:20}/DNA_F$= 1.40
Δ % R
Time (sec.)
h) Exp.8
$mix_{80:20}$
DNA_M
DNA_F
DNA_M/DNA_F= 1.28
$mix_{80:20}/DNA_F$= 1.38
Δ % R
Time (sec.)
i) Exp.9
DNA_F
DNA_M
$mix_{80:20}$
DNA_M/DNA_F=1.30
$mix_{80:20}/DNA_F$= 1.09
Δ % R
Time (sec.)
j) Exp.10
DNA_M
DNA_F
$mix_{80:20}$
DNA_M/DNA_F=1.33
$mix_{80:20}/DNA_F$= 1.27
Δ % R
Time (sec.)
k) Exp.11
DNA_M
DNA_F
$mix_{80:20}$
DNA_M/DNA_F= 1.37
$mix_{80:20}/DNA_F$=1.11
Δ % R
Time (sec.)
l) Exp.12
$mix_{80:20}$
DNA_M
DNA_F
DNA_M/DNA_F= 1.38
$mix_{80:20}/DNA_F$= 1.46
Δ % R
Time (sec.)

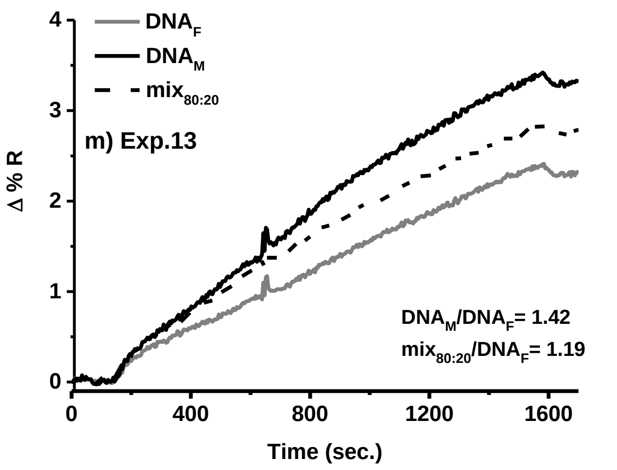
DNA$_F$
DNA$_M$
mix$_{80:20}$
m) Exp.13
Δ % R
Time (sec.)
DNA$_M$/DNA$_F$= 1.42
mix$_{80:20}$/DNA$_F$= 1.19

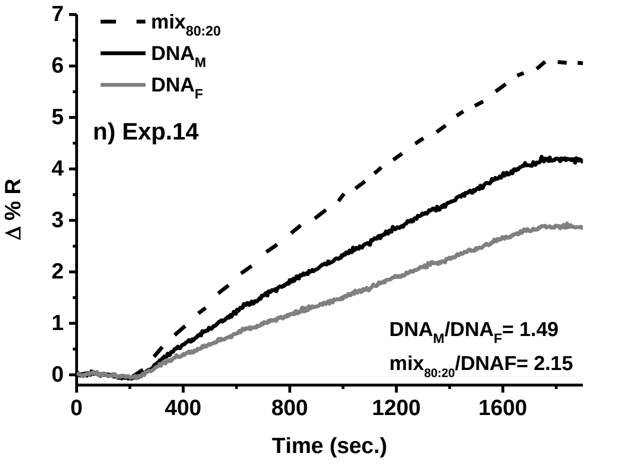
mix$_{80:20}$
DNA$_M$
DNA$_F$
n) Exp.14
Δ % R
Time (sec.)
DNA$_M$/DNA$_F$= 1.49
mix$_{80:20}$/DNAF= 2.15

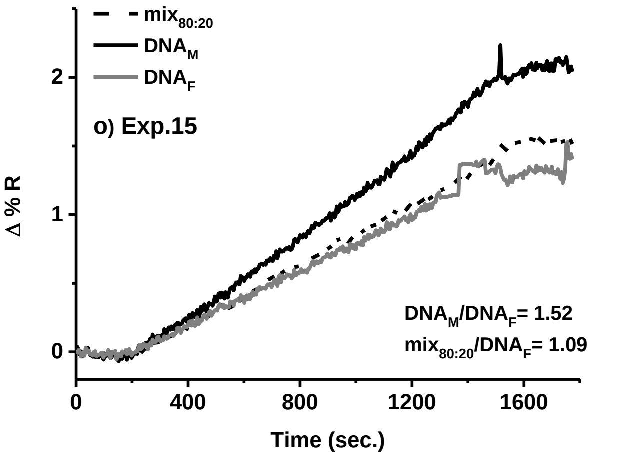
mix$_{80:20}$
DNA$_M$
DNA$_F$
o) Exp.15
Δ % R
Time (sec.)
DNA$_M$/DNA$_F$= 1.52
mix$_{80:20}$/DNA$_F$= 1.09

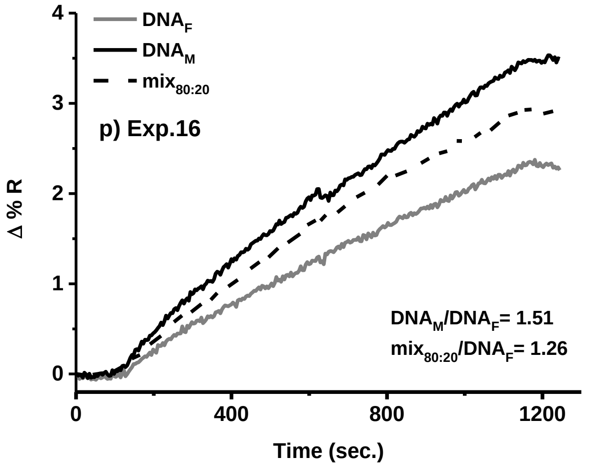
DNA$_F$
DNA$_M$
mix$_{80:20}$
p) Exp.16
Δ % R
Time (sec.)
DNA$_M$/DNA$_F$= 1.51
mix$_{80:20}$/DNA$_F$= 1.26

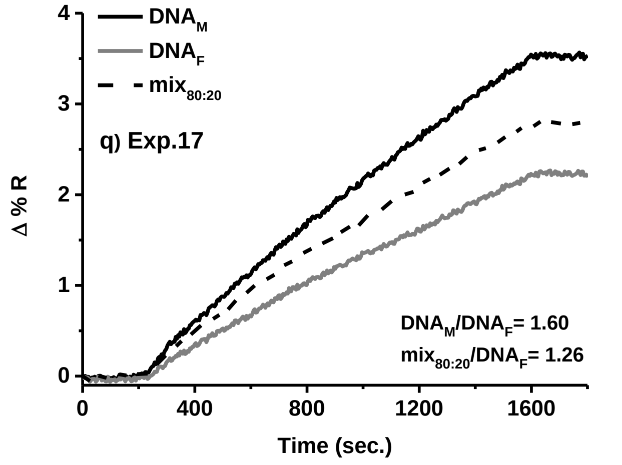
DNA$_M$
DNA$_F$
mix$_{80:20}$
q) Exp.17
Δ % R
Time (sec.)
DNA$_M$/DNA$_F$= 1.60
mix$_{80:20}$/DNA$_F$= 1.26

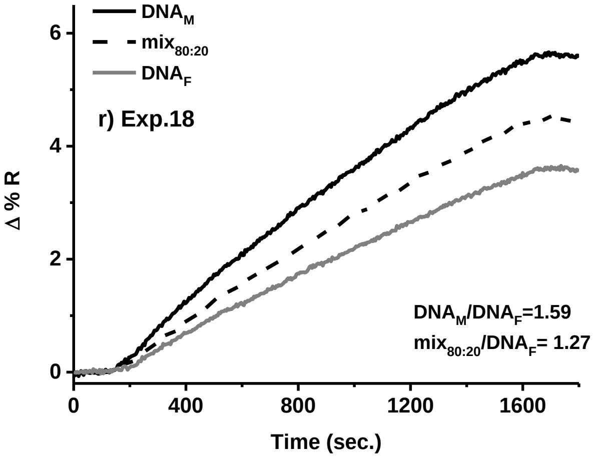
DNA$_M$
mix$_{80:20}$
DNA$_F$
r) Exp.18
Δ % R
Time (sec.)
DNA$_M$/DNA$_F$=1.59
mix$_{80:20}$/DNA$_F$= 1.27

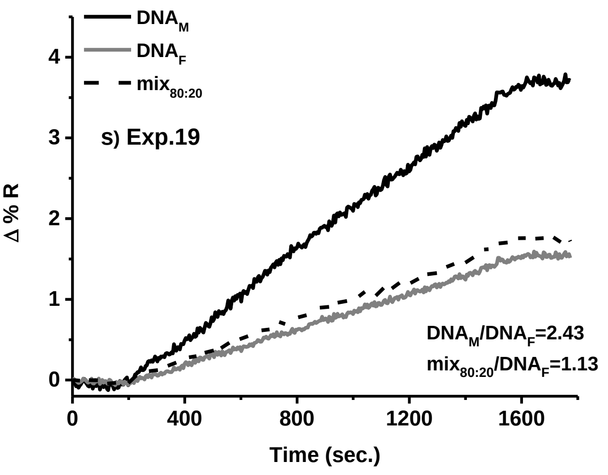
DNA_M
DNA_F
mix_80:20
s) Exp.19
Δ % R
Time (sec.)
DNA_M/DNA_F=2.43
mix_80:20/DNA_F=1.13

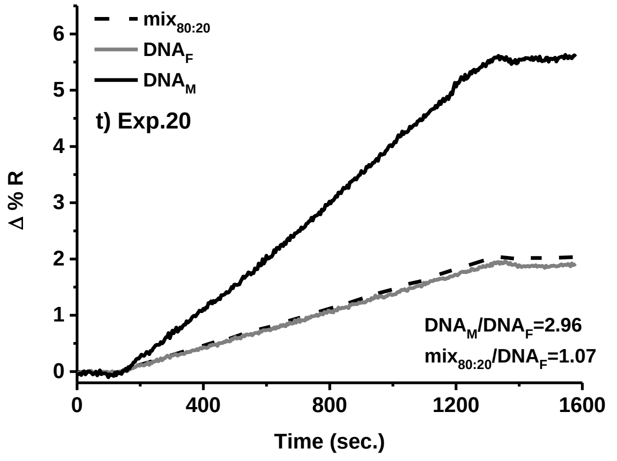
mix_80:20
DNA_F
DNA_M
t) Exp.20
Δ % R
Time (sec.)
DNA_M/DNA_F=2.96
mix_80:20/DNA_F=1.07

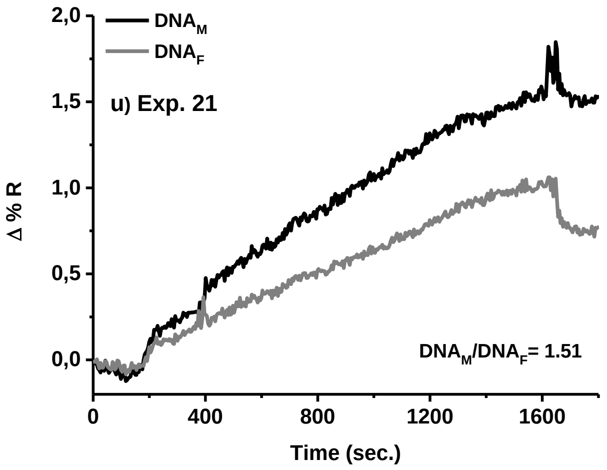
DNA_M
DNA_F
u) Exp. 21
Δ % R
Time (sec.)
DNA_M/DNA_F= 1.51

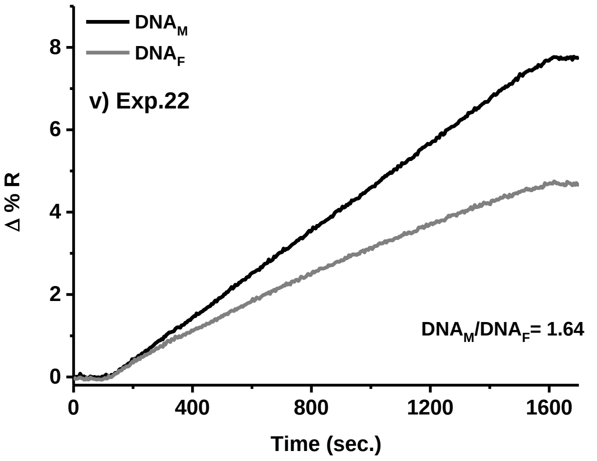
DNA_M
DNA_F
v) Exp.22
Δ % R
Time (sec.)
DNA_M/DNA_F= 1.64

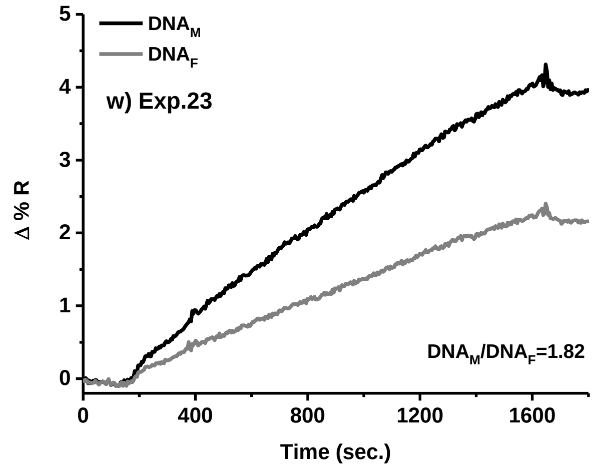
DNA_M
DNA_F
w) Exp.23
Δ % R
Time (sec.)
DNA_M/DNA_F=1.82

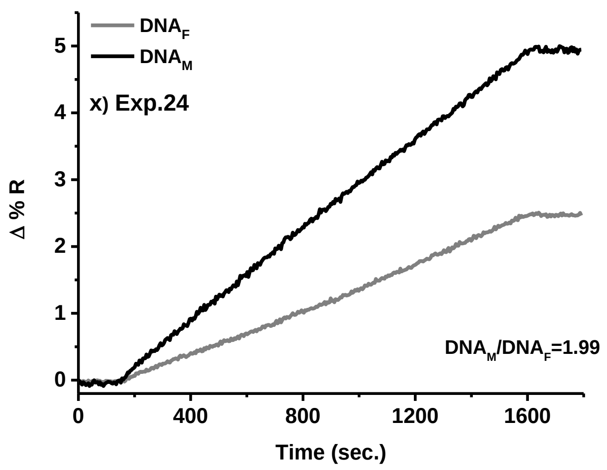
DNA_F
DNA_M
x) Exp.24
Δ % R
Time (sec.)
DNA_M/DNA_F=1.99

Figure S2: Data/box plots showing the Δ% R values for AuNP@SRY enhanced detection of $gDNA_M$, $gDNA_F$ and $mix_{80:20}$. We performed the experiments analyzing male, female and $mix_{80:20}$ genetic samples in parallel. The plot does not allow to group values resulting from each experiment. Table S1 is reported for this purpose.

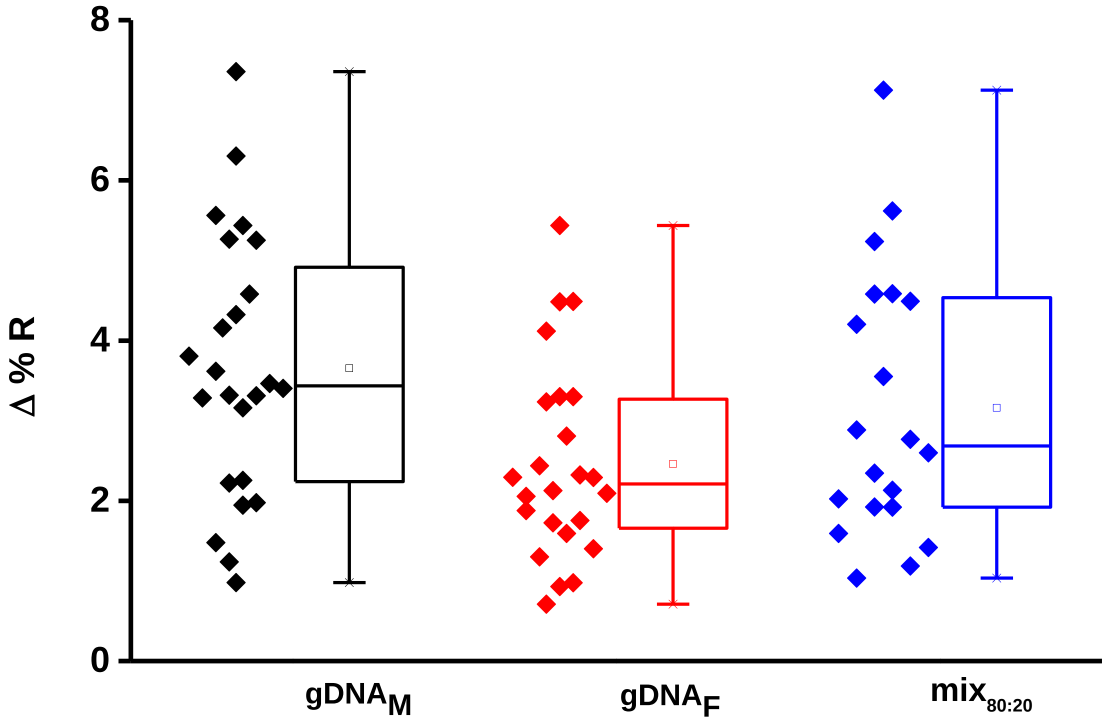

Figure S3: Box/data plots showing values of $\Delta\%RDNA_M/\Delta\%RDNA_F$ and $\Delta\%Rmix_{80:20}/\Delta\%RDNA_F$ ratios. Each couple of ratio value refers to experiments performed analyzing male, female and $mix_{80:20}$ genetic samples in parallel. A black dashed line is shown to identify ratio values larger than 1. All the performed experiments showed that the assay can discriminate between male and female gDNAs and between $mix_{80:20}$ and female gDNA (ratio value greater than 1). In addition, the medians of the two groups of ratio values are significantly different (non-parametric Kruskal−Wallis test, α=0.05, p-value= 0.0016).

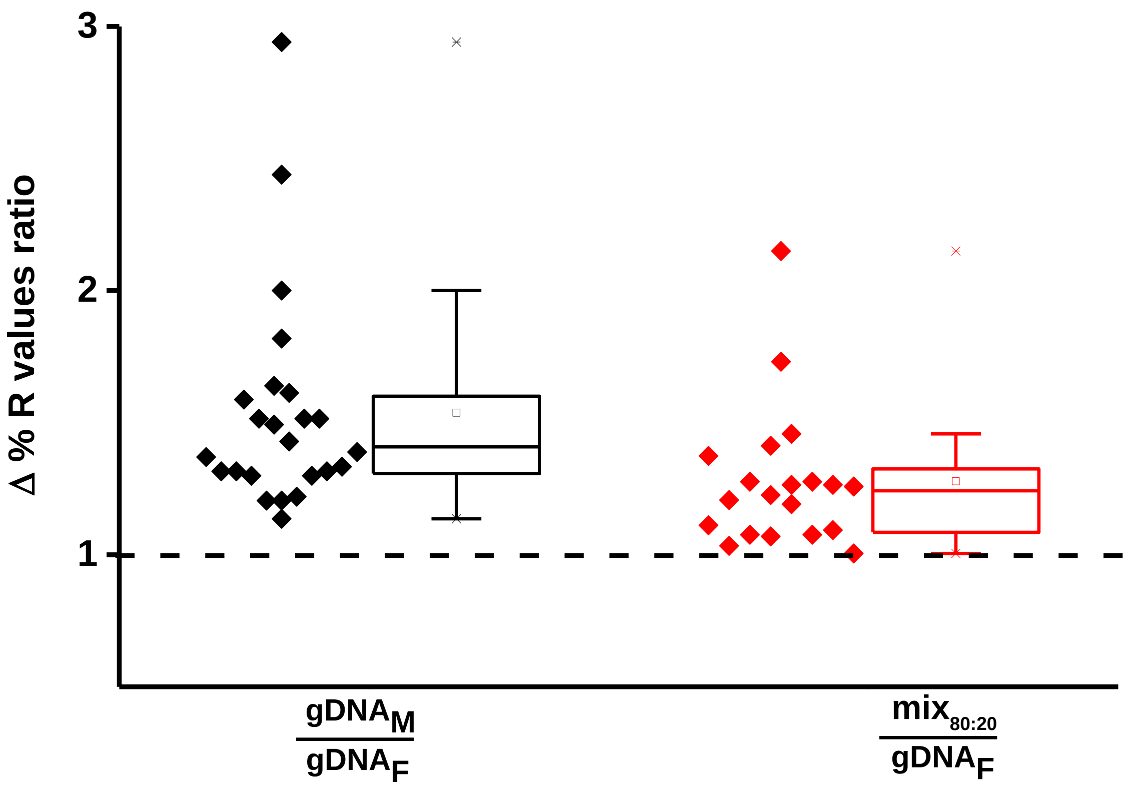

**Table S1:** Δ%R values for the AuNP@SRY enhanced parallel detection of $gDNA_M$, $gDNA_F$ and $mix_{80:20}$ samples.			
**Experiment**	**Δ%RDNA$_M$**	**Δ%RDNA$_F$**	**Δ%Rmix$_{80:20}$**
**1**	3.16194	2.80778	3.55079
**2**	5.43627	4.48466	4.58550
**3**	6.30304	5.43627	5.61874
**4**	1.94486	1.59184	1.92108
**5**	2.25561	1.72556	2.13102
**6**	5.26486	4.11759	7.12696
**7**	4.32532	3.30043	4.58122
**8**	4.15736	3.23572	4.49139
**9**	2.22208	1.75435	1.92246
**10**	1.23828	0.93010	1.18718
**11**	3.31844	2.42027	2.34590
**12**	0.97903	0.71064	1.03565
**13**	3.31154	2.32309	2.76847
**14**	3.61799	2.43644	5.23720
**15**	1.97764	1.30068	1.41817
**16**	3.46526	2.29175	2.88395
**17**	3.28523	2.05524	2.59881
**18**	5.25285	3.30043	4.20296
**19**	3.40350	1.40255	1.59184
**20**	5.56149	1.87902	2.02307
**21**	1.47862	0.97833	
**22**	7.35657	4.48751	
**23**	3.80660	2.09421	
**24**	4.58009	2.29262	

Table S2: Repeatability statistics of the ratio values		
	**Δ%RDNA$_M$ /Δ%RDNA$_F$**	**Δ%Rmix$_{80:20}$/Δ%RDNA$_F$**
Mean	1.53779 (n=24)	1.2777 (n=20)
Standard Deviation (SD)	0.41423	0.26768
CV%	27	20

Supplementary References

1) Shumaker-Parry, J. S.; Campbell, C. T. Quantitative Methods for Spatially Resolved Adsorption/Desorption Measurements in Real Time by Surface Plasmon Resonance Microscopy. *Anal. Chem.* **2004**, 76, 907−917.
2) Jung, L. S.; Campbell, C. T.; Chinowsky, T. M.; Mar, M. N.; Yee, S. S. Quantitative Interpretation of the Response of Surface Plasmon Resonance Sensors to Adsorbed Films. *Langmuir* **1998**, 14, 5636−5648.
3) Breveglieri, G.; Bassi, E.; Carlassara, S.; Cosenza, L. C.; Pellegatti, P.; Guerra, G.; Finotti, A.; Gambari, R.; Borgatti, M. Y chromosome identification in circulating cell-free fetal DNA using surface plasmon resonance, *Prenat Diagn*. **2016**, 36, 353–361.
